# Selective ERK Activation Differentiates Mouse and Human Tolerogenic Dendritic Cells, Expands Antigen-Specific Regulatory T Cells, and Suppresses Experimental Inflammatory Arthritis

**DOI:** 10.1002/art.30099

**Published:** 2011-01

**Authors:** Frederick Arce, Karine Breckpot, Holly Stephenson, Katarzyna Karwacz, Michael R Ehrenstein, Mary Collins, David Escors

**Affiliations:** 1 University College LondonLondon, UK; 2 University College LondonLondon, UKVrije Universiteit BrusselBrussels, Belgium

## Abstract

**Objective:**

Most therapeutic treatments for autoimmune arthritis rely on immunosuppressive drugs, which have side effects. Although a previous study by our group showed that specific ERK activation suppressed immune responses, its application in a therapeutic setting has never been tested. The aim of the present study was to define the ERK-dependent immunosuppressive mechanisms and to apply selective ERK activation for the treatment of experimental inflammatory arthritis.

**Methods:**

A constitutively active ERK activator was coexpressed with a model antigen using lentivectors. Immunosuppressive mechanisms were characterized at the level of dendritic cell (DC) function, differentiation of antigen-specific Treg cells, and inhibition of inflammatory T cells. Administration of the ERK activator with antigen as a strategy to suppress inflammatory arthritis was tested in an experimental mouse model.

**Results:**

Selective ERK activation induced mouse and human DCs to secrete bioactive transforming growth factor β, a process required for suppression of T cell responses and differentiation of antigen-specific Treg cells. Treg cells strongly proliferated after antigen reencounter in inflammatory conditions, and these cells exhibited antigen-dependent suppressive activities. Inflammatory arthritis was effectively inhibited through antigen-specific mechanisms. Importantly, this strategy did not rely on identification of the initiating arthritogenic antigen. Equivalent mechanisms were demonstrated in human monocyte–derived DCs, setting the scene for a possible rapid translation of this approach to patients with rheumatoid arthritis.

**Conclusion:**

This strategy of selective ERK activation resulted in an effective therapeutic protocol, with substantial advantages over DC or T cell vaccination.

Rheumatoid arthritis (RA) is an autoimmune disease that is characterized by chronic joint inflammation, which eventually leads to bone destruction and a significant decrease in life expectancy and quality of life. This disease is thought to be caused by a loss of tolerance toward autoantigens, although the specific identities of these antigens are as yet unclear. RA is currently treated with immunosuppressive drugs and biologic agents such as methotrexate and infliximab. However, these therapeutic agents induce a generalized immunosuppression that increases the risk of infectious diseases and cancer (1,2). Therefore, new therapeutic approaches should be aimed at suppression of inflammation and reestablishment of tolerance toward arthritogenic antigens without compromising the patient's immune system (1). However, the arthritogenic antigens are yet to be clearly defined, and therefore these antigen-specific therapeutic approaches should also be amenable to redirection to circumvent the need to target the pathogenic antigen. In this regard, some studies have achieved an effective approach by engineering Treg cells with well-defined specificities, in which these cells were retargeted to suppress arthritis caused by a different antigen (3). However, treatment of human patients with large numbers of modified Treg cells is a rather challenging and cumbersome process (4).

Research in immune modulation has increased in recent years, and a particular subset of dendritic cells (DCs) has been shown to modulate immune responses (5). Their suppressive activity is exerted through a variety of mechanisms, including production of immunosuppressive mediators and induction of Treg cell differentiation (6–10). Many studies have used regulatory DCs for the treatment of autoimmune disease. These DCs are generally differentiated ex vivo with a variety of tolerogenic stimuli, particularly certain Toll-like receptor and lectin ligands, immunosuppressive mediators, and cytokines (7,11–14). These ligands trigger a complex network of signaling pathways, some of which have been implicated in immune tolerance (13–17). Of particular note, the MAPK ERK has been frequently implicated in the mechanisms of immunosuppression (11,18–20), although most of the evidence has largely relied on the use of MEK chemical inhibitors rather than specific activators. Possibly as a consequence of this limitation, ERK tolerogenic activity has been questioned in a number of cases, including studies of experimental arthritis (21–23).

Manipulation of suppressive signaling pathways in combination with antigen delivery could generate potent antigen-specific tolerogenic DCs, which would prevent generalized off-target immunosuppression. For this purpose, we have developed an experimental approach to simultaneously deliver antigens and selectively activate signaling pathways, which circumvents the need for chemical inhibitors or agonists that trigger multiple signaling pathways. In this study, we evaluated in detail the role of selective ERK activation, through the use of lentivectors for the expression of constitutively active or dominant-negative mutants of MEK-1, the upstream ERK kinase, as a strategy for the treatment of inflammatory arthritis.

## MATERIALS AND METHODS

### Cells and mice

The 293T cells were grown in Dulbecco's modified Eagle's medium with 10% fetal calf serum. A transforming growth factor β (TGFβ) reporter cell line, SMAD-GFP, was engineered by transducing 293T cells with the lentivector pSIN-SMAD-GFP, as further described below. Bone marrow–derived DCs (BMDCs) were prepared from thymocyte antigen 1.2–positive (Thy1.2+) C57BL/6 and interleukin-10–knockout (IL-10^−/−^) C57BL/6 mice (24). Thy1.1+ CD57BL/6 mice and Thy1.2+ OT-I and OT-II mice, which express a transgenic T cell receptor specific for H2-K^6^–restricted class I and class II ovalbumin epitopes, respectively, were bred in-house. BMDCs were obtained using a previously described method (25). Approval for the animal studies was obtained from the University College London Animal Ethics Committee. In separate experiments, DCs prepared from human peripheral blood monocytes (PBMCs) were used for lentiviral transduction and Treg cell induction or for stimulation of CD8+ T cells, as described previously (26,27).

### Plasmids, lentivector production, titration, and cell transduction

Dual lentivectors coexpressing MEK-1 mutants along with green fluorescent protein (GFP) or invariant-chain ovalbumin (IiOVA) were used (25). The hemagglutinin tag was attached to the IiOVA C-terminus. A lentivector expressing GFP under the control of a promoter responsive to TGFβ signaling (28) was constructed (pSIN-SMAD-GFP). A lentivector short hairpin RNA (shRNA) delivery system was engineered by introducing a microRNA miR-30 sequence into a synthetic chimeric intron derived from the plasmid pHygEGFP (Clontech). TGFβ shRNA were selected using the Hannon Laboratory design tool (see http://hannonlab.cshl.edu/GH_shRNA.html). The shRNA in the miR-30 sequence were introduced within the synthetic intron, and this was then inserted in the lentivector plasmid pDUAL-MEK-1 ED-IiOVA. The sequence of the shRNA used in the present experiments (designated TGFβ-C) was 5′-TGCTGTTGACAGTGAGCGACGGCAGCTGTACATTGACTTTTAGTGAAGCCACAGATGTAAAAGTCAATGTACAGCTGCCGGTGCCTACTGCCTCGGA-3′. An inactive CD40-targeted shRNA (designated CD40-B) was selected as the untargeted control, and its sequence was 5′-TGCTGTTGACAGTGAGCGAACAGACACTGTGAACCCAATCTAGTGAAGCCACAGATGTAGATTGGGTTCACAGTGTCTGTGTGCCTACTGCCTCGGA-3′.

Lentivectors were produced and titrated by quantitative polymerase chain reaction, as described previously (29). Mouse BMDCs and human monocyte–derived DCs were transduced using previously described methods (25–27,29). A multiplicity of transduction between 5 and 30 was used to modify the DCs.

### Cell staining and flow cytometry

Surface and intracellular staining were performed as described previously (25). Stained cells were analyzed in a Becton Dickinson LSR flow cytometer and FACSCalibur. The following anti-mouse antibodies were purchased from eBioscience, together with the appropriate isotype controls: biotinylated antibodies specific for Thy1.1 and Thy1.2, phycoerythrin (PE)–conjugated anti-CD25, anti-CD8, PE-Cy7–conjugated anti-CD4, allophycocyanin (APC)–conjugated anti-FoxP3, fluorescein isothiocyanate (FITC)–conjugated anti–IL-10, APC-conjugated anti–interferon-γ (anti-IFNγ), and FITC-conjugated anti–IL-17A. Human PE-Cy7–conjugated anti-CD4 and APC-conjugated anti-CD8 were purchased from BD PharMingen. Human PE-conjugated CD25 and APC-conjugated anti-FoxP3 were purchased from Miltenyi Biotec and eBioscience, respectively.

### Vaccinations and T cell responses

All vaccination experiments were repeated independently at least 3 times, using groups of 5 mice that were vaccinated as previously described (25). For DC vaccination, mice were vaccinated subcutaneously, as described previously (25). When appropriate, DCs were incubated with increasing concentrations of the inhibitors U0126 and PD98059 (Cell Signaling Technology).

Class I OVA–specific enzyme-linked immunospot (ELISpot) assays for IFNγ were performed as described previously (25). Stainings for the OVA-specific class I pentamer within CD3+CD8+ T cells were performed according to the manufacturer's protocol (Proimmune).

### Murine antigen-induced arthritis (AIA)

Induction of arthritis with methylated bovine serum albumin (mBSA), histologic staining of mouse knee sections with hematoxylin and eosin, and scoring of histologic features of murine AIA were performed as previously reported (3). Groups of 5 mice were vaccinated with lentivectors, and AIA was initiated (3). Knee sections were visualized using a Nikon Eclipse TS100 microscope with a 40×/0.55 magnification objective, and images were obtained with a Nikon E950 digital camera. Histologic scoring for the extent of joint destruction and inflammation was performed in a blinded manner, using a scale of 0–3. A combined score was generated from the sum of the individual scores (maximum arthritis score of 6).

### T cell purification and antigen-linked suppression assay

CD4+ T cells were purified from mouse spleens using the Dynal Mouse CD4 Negative Isolation Kit (Invitrogen), and CD4+CD25+ T cells were isolated using the CD4+CD25+ regulatory T cell isolation kit (Miltenyi Biotec). Human naive CD4+CD25− T cells and CD8+ T cells were isolated from human PBMCs obtained from at least 6 different donors (30). An antigen-linked suppression assay was performed as described previously (3), using carboxyfluorescein succinimidyl ester (CFSE)–labeled OVA-specific OT-I CD8+ cells as targets. Briefly, splenocytes from the OT-I–transgenic mouse strain were labeled with CFSE, as described by the manufacturers (Invitrogen). Splenocytes were pulsed with 0.1 ng/μl class I OVA peptide (SINKFELL). Increasing numbers of purified CD4+CD25+ T cells were added, and class II OVA_323–339_ peptide was added at 2 μg/ml when indicated. A peptide from the hepatitis B virus core was used as a control (31). Cell proliferation was analyzed with FlowJo software.

Allogeneic cocultures of human DCs with naive human T cells were set up, and Treg cells were harvested and analyzed after 3 days as previously described (30). An autologous suppression assay with matured human DCs and purified Treg cells was performed as described previously (32), using sorted CD8+ T cells as targets, in the absence or presence of anti-CD3/anti-CD28 beads. Autologous sorted CD25^high^ T cells were added at increasing ratios, and proliferation of the cells was measured by determination of ^3^H-methyl-thymidine uptake (Amersham) (32).

### Statistical analysis

To analyze the ELISpot data, normality was checked using the Kolmogorov-Smirnov test (25). Pairwise comparisons for posterior probability were performed using Tukey's test. A minimum of 3 independent experiments was performed with 5 mice per group. To compare the mean fluorescence intensity from surface or intracellular staining, with values adjusted for interexperimental variability, 2 different approaches were undertaken. In the first approach, data were normalized relative to the value for control untreated cells, and comparisons were performed as described above. In the second approach, groups were compared using raw data in a two-way analysis of variance with a random criterium (experiments), and pairwise comparisons for posterior probability were performed when required (25). Data from antigen-linked suppression assays and histologic scorings were analyzed as described previously (3).

## RESULTS

### Suppression of effector T cell expansion by selective ERK activation, leading to antigen-specific FoxP3+ Treg cell differentiation

**Figure 1:**
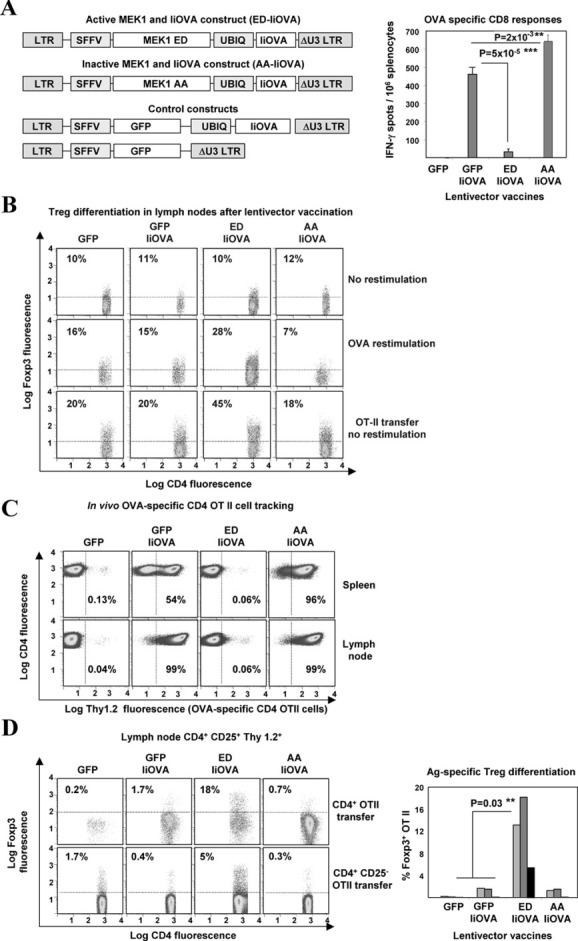
ERK activation prevents T cell expansion and differentiates FoxP3+CD4+ T cells. **A**, Left, Schemes of lentiviral constructs coexpressing MEK-1 ED (for active MEK-1) or MEK-1 AA (for inactive MEK-1) with the class II major histocompatibility complex fusion antigen invariant-chain ovalbumin (IiOVA), or control constructs expressing green fluorescent protein (GFP) with or without IiOVA. Right, Ovalbumin (OVA)–specific CD8 T cell responses, as determined by interferon-γ (IFNγ) enzyme-linked immunospot assay, in mouse spleens after lentivector vaccination. Bars show the mean ± SD results in 5 mice per group. **B**, FoxP3 expression in lymph node CD4+CD25+ T cells after lentivector vaccination, without or with OVA restimulation and after intravenous transfer of OVA-specific CD4+ OT-II cells without restimulation. Values are the percentage of FoxP3+ T cells. **C**, Thymocyte antigen 1.2 (Thy1.2) expression in lymph node CD4+ T cells from Thy1.1+ mice that received transferred Thy1.2+ OT-II cells. Values are the percentage of Thy1.2+ T cells. **D**, Left, FoxP3 expression from lymph node Thy1.2+CD4+CD25+ T cells in lentivector-vaccinated Thy1.1+ mice after transfer of CD4+ OT-II cells or CD4+CD25− OT-II cells. Values are the percentage of FoxP3+ T cells. Right, FoxP3 expression in OT-II cells after lentivector vaccination in 3 independent experiments (each represented as a differently shaded bar). Horizontal or vertical broken line indicates the cutoff for positivity. ∗∗ = very significant difference (*P* < 0.01); ∗∗∗ = highly significant difference (*P* < 0.001). LTR = long terminal repeat; SFFV = spleen focus–forming virus; UBIQ = ubiquitin; Ag = antigen.

Autoimmune disorders such as rheumatoid arthritis are caused by uncontrolled immune responses toward autoantigens, a process that leads to acute and chronic joint inflammation. For the treatment of these disorders, effective immunosuppressive therapies are necessary for the suppression of T cell responses and Treg cell differentiation (1,3). We have previously shown, using dual lentivectors, that coexpression of a constitutively active MEK-1 mutant (MEK-1 ΔNES ED, or active MEK-1) with the model class II major histocompatibility complex fusion antigen IiOVA (MEK-1 ED–IiOVA construct) (Figure 1A) will lead to strong inhibition of T cell responses and, possibly, to Treg cell differentiation (25,33).

In this study, we wanted to selectively activate ERK for the treatment of inflammatory arthritis. First, we confirmed that a highly significant reduction of OVA-specific CD8 T cells was achieved only when active MEK-1 and IiOVA were simultaneously expressed from the same lentivector backbone, with a reduction from 500 spots per million splenocytes down to ∼40 spots per million splenocytes, as assessed by IFNγ ELISpot assay (Figure 1A). Interestingly, OVA-specific CD8 responses were significantly boosted when the ERK activator was replaced by an inactive version (MEK-1 ΔNES AA, or inactive MEK-1) (Figure 1A).

Our data also suggested that antigen-specific Treg cell differentiation was taking place when the ERK activator and IiOVA were codelivered (25,33). To demonstrate Treg cell differentiation, groups of congenic Thy1.1+ mice were vaccinated with each lentivector. One day later, 10^6^ purified OVA-specific CD4+Thy1.2+ OT-II T cells per mouse were transferred intravenously, and FoxP3 expression was assessed in the OT-II cells from the draining inguinal lymph nodes. In the absence of OT-II cell transfer, FoxP3+ T cell expansion was observed only after ex vivo OVA restimulation in mice vaccinated with the ERK activator–IiOVA (Figure 1B). In contrast, when OT-II cells were transferred, increased numbers of FoxP3+ Treg cells were evident in the lymph nodes, without the need for OVA restimulation (Figure 1B).

Quantification of Thy1.2+ T cells in the lymph nodes and spleens of mice suggested that there was a lack of significant proliferation only when the ERK activator and IiOVA were coexpressed (Figure 1C). In contrast, virtually all CD4+ T cells were observed to be Thy1.2+ in mice vaccinated with lentivectors expressing GFP-IiOVA or inactive MEK-1–IiOVA (Figure 1C). Moreover, only the coexpression of active MEK-1 with IiOVA resulted in a significant increase in FoxP3+ expression within CD4+CD25+Thy1.2+ OT-II cells (Figure 1D). The same ERK-dependent FoxP3 induction was observed after transfer of 3 × 10^6^ purified naive CD25− OT-II cells, albeit at lower levels (Figure 1D). Overall, these results indicate that ERK activation prevented antigen-specific effector T cell expansion, resulting in antigen-specific CD4+CD25+FoxP3+ Treg cell differentiation.

### Strong boosting of antigen-specific FoxP3+ Treg cell expansion after a second antigen encounter under inflammatory conditions

Treg cells are particularly effective in inhibiting arthritis in mouse models (3). Therefore, we tested whether these differentiated Treg cells could expand in vivo after a second antigen encounter, a strategy that could be exploited to prevent arthritis. Mice primed with lentivectors were rechallenged with purified OVA either in phosphate buffered saline (PBS) as control or in Freund's incomplete adjuvant (IFA). Four days later, Treg cells were quantified in the draining lymph nodes.

**Figure 2:**
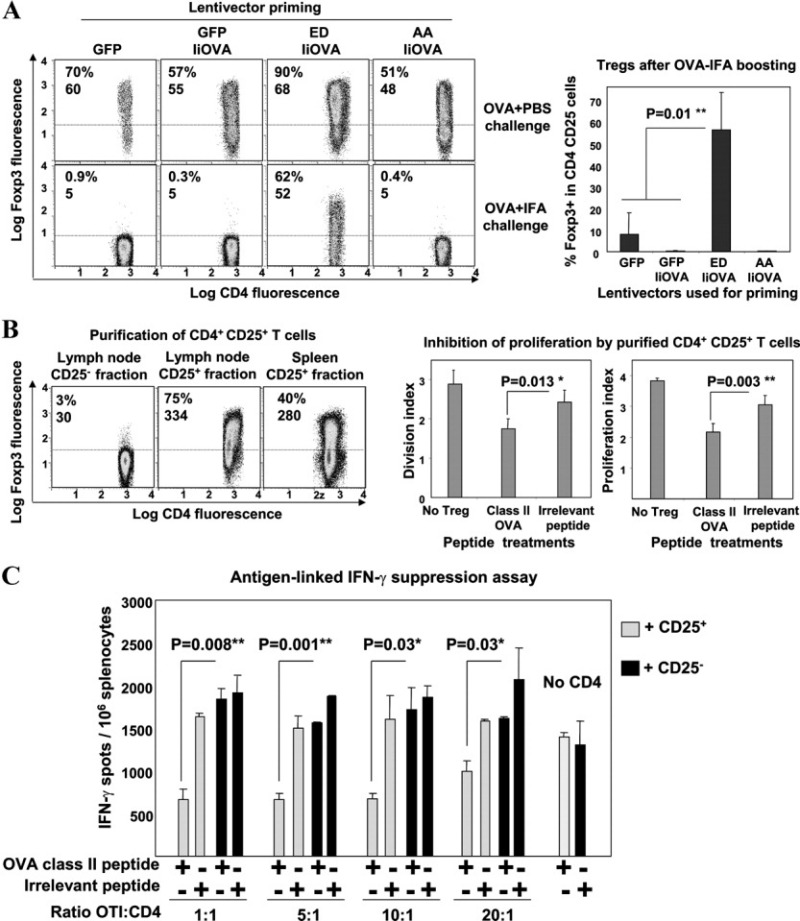
Antigen-specific FoxP3+ Treg cells undergo expansion after antigen reencounter. **A**, Left, FoxP3 expression in lymph node CD4+CD25+ T cells following lentivector vaccination (control constructs expressing GFP or GFP plus IiOVA, and constructs with active MEK-1 [ED] or inactive MEK-1 [AA] plus IiOVA) and rechallenge with OVA plus phosphate buffered saline (PBS) or Freund's incomplete adjuvant (IFA). Values are the percentage and mean fluorescence intensity (MFI) of FoxP3 expression. Right, Expression of FoxP3+ cells in lymph node Treg cells after lentivector priming and OVA plus IFA boosting. **B**, Left, FoxP3 expression in purified lymph node CD4+CD25+ T cells or spleen CD4+CD25+ T cells after active MEK-1–IiOVA priming and OVA plus IFA boosting. Values are the percentage and MFI of FoxP3 expression. Right, Inhibition of T cell proliferation according to treatment group (no Treg cells, class II OVA peptide, or irrelevant peptide), as measured by the division index and proliferation index. In **A** and **B**, the horizontal broken line indicates the cutoff for positivity. **C**, CD8+ IFNγ responses in peptide-pulsed OT-I mouse splenocytes incubated with purified Treg cells at the indicated ratios. A class II OVA peptide or an irrelevant peptide was present (+) or absent (−) in the assays with CD25+ or CD25− cells. Bars in **A–C** show the mean ± SEM results in 5 mice per group. ∗ = very significant difference (*P* < 0.01); ∗∗ = highly significant difference (*P* < 0.001). See Figure 1 for other definitions.

Although FoxP3+ T cell expression increased in all vaccination groups challenged with OVA in PBS (Figure 2A), only priming with the ERK activator IiOVA resulted in vigorous endogenous FoxP3+ Treg cell expansion when OVA was administered under inflammatory conditions (OVA plus IFA) (Figure 2A). ERK priming/OVA plus IFA boosting regularly resulted in 70–75% of FoxP3+ cells in CD4+CD25+ T cells purified from the draining lymph nodes, but not in those from the spleens (Figure 2B).

The suppressive activities of these cells were tested in an antigen-linked suppression assay (3). CFSE-labeled splenocytes from transgenic OT-I mice (OVA-specific Thy1.2+ CD8+ T cells) stimulated with class I OVA peptide were used as suppression targets. Purified Thy1.1+ Treg cells inhibited CD8+ OT-I cell proliferation only when the class II OVA peptide was present, as assessed by CFSE dilution after 3 days (Figure 2B). A significant decrease was observed for both the division index (representing the average number of cell divisions from responding cells) and the proliferation index (representing the average number of cell divisions that a cell in the original population has undergone) (Figure 2B). No significant inhibition was observed in the presence of an irrelevant peptide or when the CD4+CD25− counterparts were used (results not shown).

Treg cell suppressive activity toward IFNγ responses was also tested by ELISpot assay after a 16-hour incubation period. Interestingly, CD8+ T cell IFNγ responses were significantly inhibited by purified Treg cells at all tested ratios only in the presence of class II OVA peptide (Figure 2C). These results indicate that Treg cells that expanded after antigen reencounter exerted antigen-specific inhibitory activities toward CD8+ T cell proliferation and IFNγ responses.

### Modulated DC function and differentiation of tolerogenic DCs with TGFβ-dependent suppressive activities after selective ERK activation

Subcutaneous vaccination with lentivectors results in transduction of DCs that subsequently migrate to draining lymph nodes and present antigen to T cells (34,35). We hypothesized that DCs may be directly involved in ERK-dependent immunosuppression, which could lead to differentiation of immunosuppressive Treg cells. To characterize the DC-dependent mechanisms involved in ERK tolerogenicity, we tested the capacity of ERK-activated BMDCs to suppress in vivo immune responses.

**Figure 3:**
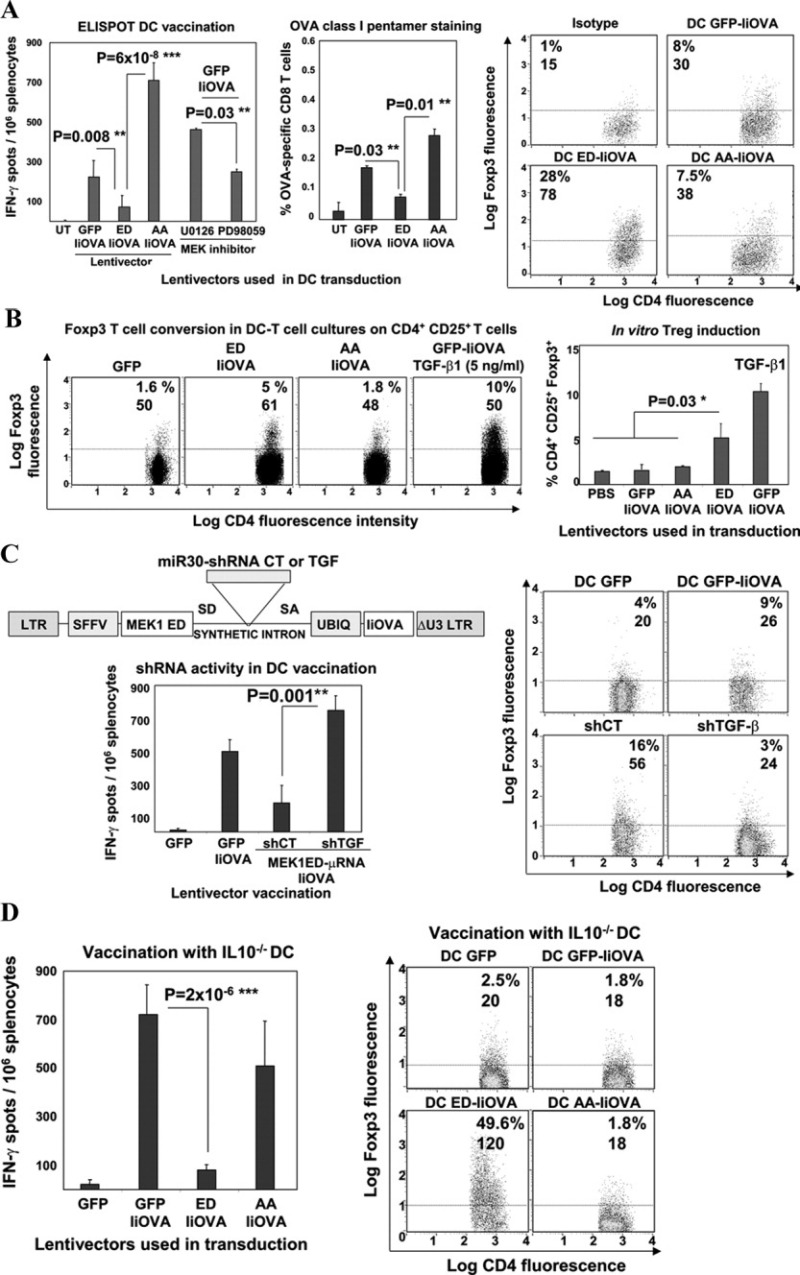
ERK-dependent immunosuppression is dependent on dendritic cell (DC)–derived transforming growth factor β (TGFβ). **A**, Left, CD8 T cell responses, as determined by IFNγ enzyme-linked immunospot (ELISpot) assay or OVA class I pentamer staining, after vaccination with lentivector-modified DCs (or untransduced [UT] DCs as control) or after MEK inhibitor treatments. Right, FoxP3 expression in splenocyte CD4+CD25+ T cells after lentivector vaccination. Values are the percentage and mean fluorescence intensity (MFI) of FoxP3+ expression. **B**, Left, FoxP3 expression in CD4+CD25+ OT-II cells cultured with transduced DCs. Values are the percentage and MFI of FoxP3 expression. Right, In vitro Treg cell induction of FoxP3 expression in the DC–T cell cocultures, without or with TGFβ1 treatment at 5 ng/ml. **C**, Left (top), Scheme of the lentivector short hairpin RNA (shRNA) delivery platform, highlighting the intron with microRNA miR-30 shRNA. Left (bottom), CD8+ T cell responses, as determined by IFNγ ELISpot assay, after vaccination with ex vivo lentivector-modified DCs and codelivery with TGFβ-targeted shRNA (shTGF) or control shRNA (shCT). Right, FoxP3 expression in splenocyte CD4+CD25+ T cells after vaccination with lentivector-modified DCs or shRNA, followed by OVA restimulation. Values are the percentage and MFI of FoxP3+ expression. **D**, Left, OVA-specific CD8 responses, as determined by IFNγ ELISpot assay, in mice vaccinated with lentivector-transduced interleukin-10–knockout (IL-10^−/−^) DCs. Right, FoxP3 expression in splenocyte CD4+CD25+ T cells vaccinated with transduced IL-10^−/−^ DCs. Values are the percentage and MFI of FoxP3+ expression. Horizontal broken lines indicate the cutoff for positivity. Bars in **A–D** show the mean ± SEM. ∗ = significant difference (*P* < 0.05); ∗∗ = very significant difference (*P* < 0.01); ∗∗∗ = highly significant difference (*P* < 0.001). PBS = phosphate buffered saline; SD = splicing donor site; SA = splicing acceptor site (see Figure 1 for other definitions).

ERK activation in DCs significantly decreased OVA-specific CD8+ T cell responses and expanded FoxP3+ Treg cells, while ERK inhibition increased responses (Figure 3A). Similarly, DCs expressing IiOVA and treated with the MEK chemical inhibitor U0126, but not those treated with the inhibitor PD98059, had significantly increased CD8 responses (Figure 3A). These results confirm that ERK-activated DCs are immunosuppressive, while expression of the MEK-1 dominant-negative mutant enhances their immunogenicity.

The capacity of ERK-activated DCs to directly expand antigen-specific Treg cells from OT-II cells was also tested. Interestingly, the expression of CD4+CD25+FoxP3+ OT-II cells increased only when active MEK-1–IiOVA was expressed or when IiOVA-expressing DCs were treated with TGFβ, a well-known FoxP3 inducer (36,37) (Figure 3B). No conversion to FoxP3+ Treg cells was observed in CD4+ T cells from nontransgenic mice (results not shown). These results indicate that ERK-activated DCs expanded FoxP3+ T cells from antigen-specific T cells, although the effect was less efficient than that after in vivo lentivector administration of the ERK activator.

Previously, we have shown that selective ERK activation in DCs increased TGFβ expression (25). We functionally confirmed these results by engineering a 293T cell line containing a well-characterized TGFβ-responder promoter (28) driving GFP expression (SMAD-GFP 293T cells) (results not shown). TGFβ bioactivity was evident in supernatants from ERK-activated BMDCs (in the order of 200 pg/million transduced DCs), in contrast to the supernatants from inactive MEK-1– or GFP-expressing DCs (<10 pg/million transduced DCs) (results not shown). To clarify the contribution of TGFβ to ERK-induced immunosuppression, we developed a lentivector-based codelivery platform of TGFβ-targeted shRNA with active MEK-1 and IiOVA (Figure 3C). Modified DCs were administered to naive mice, and it was found that delivery of a TGFβ-specific shRNA, but not a control shRNA, completely abrogated the ERK-mediated CD8+ T cell suppressive activity and FoxP3+ Treg cell induction (Figure 3C).

We previously have shown that ERK-activated DCs in culture did not increase IL-10 secretion (25), although the possibility cannot be discarded that DC-derived IL-10 may still play a role in vivo. Interestingly, selective ERK activation in IL-10^−/−^ BMDCs very efficiently suppressed CD8+ T cell responses and induced expression of FoxP3+ T cells (Figure 3D). These results suggest that DC-induced immunosuppression by ERK activation was dependent on TGFβ bioactivity, but independent of IL-10.

### Generation of regulatory human DCs by selective ERK activation

**Figure 4:**
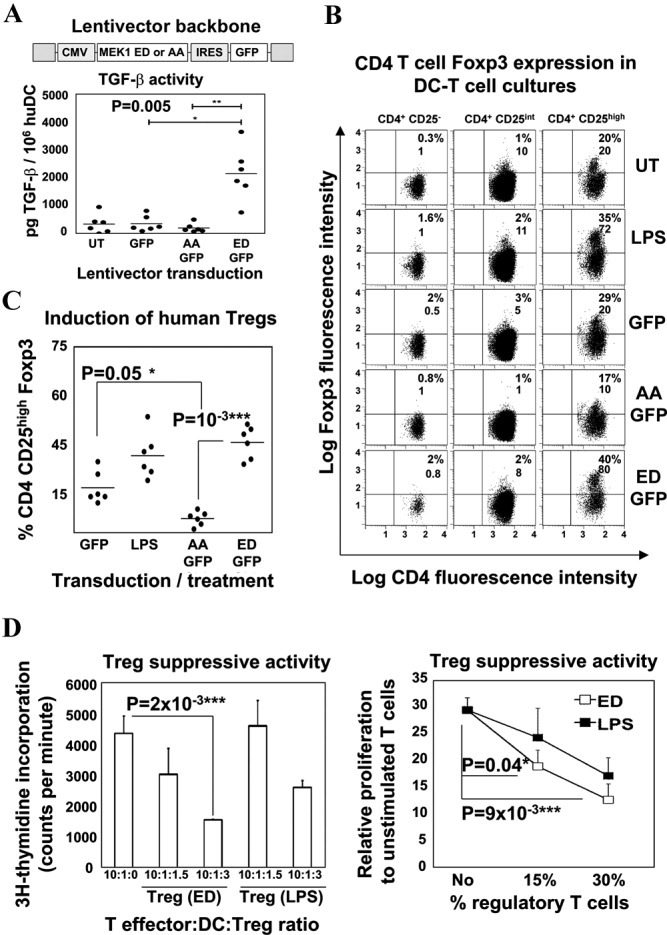
Selective ERK activation differentiates regulatory human monocyte–derived dendritic cells (huDC). **A**, Top, Construct with the active (ED) or inactive (AA) MEK-1 and the indicated cytomegalovirus (CMV) promoter and internal ribosome entry site (IRES) leading to GFP expression. Bottom, Transforming growth factor β (TGFβ) activity in supernatants of transduced huDC cultures, as assessed in SMAD-GFP cells. **B**, FoxP3 expression, as assessed by flow cytometry, in T cells from allogeneic huDC–naive CD4+ T cell cocultures. DCs were left untransduced (UT) or were transduced with the various lentivectors. T cells were gated according to CD25 expression (CD25−, CD25^intermediate^ [CD25^int^], and CD25^high^). Values are the percentage and mean fluorescence intensity (MFI) of FoxP3+ expression. LPS = lipopolysaccharide-matured DCs (positive control). **C**, Percentage of induced CD4+CD25+FoxP3+ T cell expression in 6 different donor combinations (solid circles) using transduced DCs with the indicated lentivectors. In **A** and **C**, horizontal bars show the mean. **D**, Left, Proliferation of target CD8 T cells in a representative experiment, as a function of the proportion of Treg cells purified from the same cultures as described in **B**, with results expressed as level of ^3^H-thymidine incorporation. Right, Proliferation of CD8+ T cells in response to stimulation with various percentages of Treg cells, relative to that in unstimulated T cells. Bars show the mean ± SEM data from 3 independent experiments. LPS = T cells purified from LPS-treated huDC/T cell cocultures; ED = T cells purified from ERK-activated huDC/T cell cocultures. ∗ = significant difference (*P* < 0.05); ∗∗ = very significant difference (*P* < 0.01); ∗∗∗ = highly significant difference (*P* < 0.001). See Figure 1 for other definitions.

Our results in murine cells demonstrated that ERK activation resulted in differentiation of regulatory DCs with immunosuppressive capacity, leading to Treg cell expansion. To find out whether equivalent mechanisms are present in human cells, we next tested whether ERK activation could differentiate regulatory DCs from human PBMC–derived DCs. The demonstration of this point is of particular importance for the application of our therapeutic strategy to patients with RA. In these experiments, we used lentivectors encoding ERK modulators under the control of a cytomegalovirus promoter followed by an internal ribosome entry site leading to GFP expression (Figure 4A). These lentivectors were used because the spleen focus–forming virus and ubiquitin promoters were found to be particularly inefficient in human PBMC–derived DCs.

ERK-activated human PBMC–derived DCs exhibited an immature phenotype (results not shown). In addition, high levels of TGFβ bioactivity were evident, as assessed in SMAD-GFP cells, only when active MEK-1 was expressed (Figure 4A), while no changes in the levels of IL-6, IL-12p70, and IL-10 were observed (results not shown).

To test the capacity of ERK-activated human PBMC–derived DCs to differentiate Treg cells from naive T cells, we used allogeneic DC–T cell cocultures to circumvent the need for specific antigen recognition. A significant increase in the expression of CD4+CD25^high^FoxP3+ T cells as well as in FoxP3 expression was evident only when human PBMC–derived DCs were expressing the ERK activator, while a decrease was observed when the inactive MEK-1 mutant was used (Figures 4B and C); as a positive control, lipopolysaccharide-treated human PBMC–derived DCs were used to induce Treg cell differentiation, as described previously (38).

To eliminate the possibility that FoxP3+ T cells generated by selective ERK activation were effector cells, their suppressive capacity was tested utilizing an autologous suppression assay (30,32). Briefly, matured human PBMC–derived DCs and sorted CD8+ T cells from a single donor were cocultured in the presence of anti-CD3/anti-CD28 beads. Sorted CD4+CD25^high^ T cells purified from ERK-activated human PBMC–derived DC–T cell cocultures significantly inhibited CD8+ T cell proliferation, as measured by ^3^H-thymidine incorporation (Figure 4D). Therefore, ERK-activated human PBMC–derived DCs induced functional CD4+CD25+FoxP3+ T cell differentiation, leading to suppressive activities.

### Prevention of AIA without targeting the arthritogenic antigen after in vivo administration of the ERK activator

**Figure 5:**
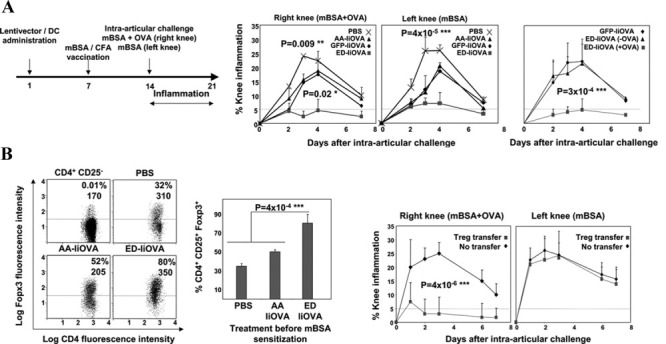
In vivo delivery of the ERK activator effectively inhibits murine antigen-induced arthritis without targeting the arthritogenic antigen. **A**, Left, Experimental vaccination regimen used to induce arthritis. **Arrows** at the top indicate the day on which each step took place. Middle, Extent of inflammation after intraarticular challenge with methylated bovine serum albumin (mBSA) in the presence (right knee) or absence (left knee) of OVA, following vaccination with the constructs containing phosphate buffered saline (PBS), the inactive (AA) or active (ED) ERK forms with IiOVA, or GFP with IiOVA. Right, Extent of inflammation in experiments with the ERK activator (ED) after intraarticular challenges performed in the absence (−) or presence (+) of OVA. **B**, Left, FoxP3 expression in CD4+CD25+ T cells, as assessed by flow cytometry, from draining lymph nodes of mice after vaccination with the indicated lentivectors and induction of antigen-induced arthritis (AIA). Analyses were performed on day 4 postchallenge. Values are the percentage and mean fluorescence intensity (MFI) of FoxP3+ expression. Middle, FoxP3 expression in CD4+CD25+ T cells from draining lymph nodes after lentivector vaccination and induction of AIA. Right, Extent of knee inflammation after OVA-specific Treg cell transfer to mBSA-sensitized mice before challenge. Bars show the mean ± SEM. The horizontal broken lines indicate the cutoff for positivity. ∗ = significant difference (*P* < 0.05); ∗∗ = very significant difference (*P* < 0.01); ∗∗∗ = highly significant difference (*P* < 0.001). CFA = Freund's complete adjuvant (see Figure 1 for other definitions).

Our results showed that selective ERK activation was clearly immunosuppressive, leading to differentiation of regulatory DCs and Treg cells. We next applied our strategy to a well-established inflammatory arthritis model in mice, the AIA model (3). This model relies on sensitization with mBSA, followed by intraarticular challenge to the knee joint with mBSA in the presence (right knee) or absence (left knee) of the tolerizing protein. Direct lentivector administration or DC vaccination was applied before mBSA sensitization (Figure 5A). Although DC vaccination yielded similar results, we chose to focus on direct lentivector administration in further studies, due to its simplicity.

The extent of inflammation, which was measured as the percent change in knee swelling, was significantly reduced (mean values lower than 5%) only when the ERK activator and IiOVA were coadministered, but this did not occur in control groups, such as delivery of the ERK inhibitor with IiOVA (Figure 5A). The ERK-targeted antigen (OVA) caused this therapeutic effect, because it was only observed when OVA was coinjected with mBSA (Figure 5A). This result demonstrated that antigen-specific tolerance was achieved, and excluded the possibility of off-target tolerization toward mBSA. Interestingly, inhibition of inflammation was also observed in the contralateral knee (challenge with mBSA alone) (Figure 5A).

Strong FoxP3+ Treg cell proliferation was observed in the draining lymph nodes only after treatment with the ERK activator and IiOVA (Figure 5B), which is consistent with our earlier results (Figure 2). To test whether Treg cells were responsible for the therapeutic effects, 10^6^ purified OVA-specific Treg cells per mouse (Figure 2B) were intravenously transferred before the induction of AIA. Transfer of OVA-specific Treg cells strongly inhibited knee inflammation when OVA was provided along with mBSA (Figure 5B). Interestingly, the therapeutic effects in the contralateral knee were lost (Figure 5B), suggesting that additional suppressive mechanisms might take place after direct lentivector administration of the ERK activator. In addition to Treg cell expansion, selective ERK activation strongly reduced the expression of inflammatory T cell subsets, particularly IFNγ+IL-17+ double-positive cells, the expression of which was completely abrogated (results not shown).

**Figure 6:**
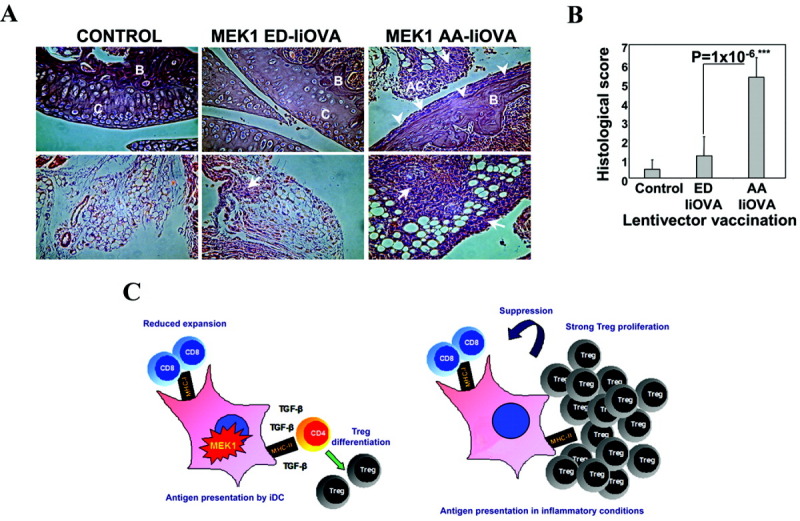
Prevention of joint destruction by delivery of the ERK activator (**A** and **B**), and working model for ERK-dependent immunosuppression (**C**). **A**, Photomicrographs show hematoxylin and eosin–stained mouse knee joint sections after challenge with methylated bovine serum albumin (mBSA) and OVA. Top panels, Articular cartilage and bone sections. Bottom panels, Articular capsule. In the control group (intraarticular injection with mBSA + OVA in naive mice), the joints show an absence of inflammation and a lack of cellular infiltration. In the group vaccinated with the ERK activator and IiOVA (MEK1 ED–IiOVA) before induction of antigen-induced arthritis (AIA), the integrity of the articular cartilage and bone is evident, with limited cellular infiltration in the articular capsule (**arrow**). In the group vaccinated with the inactive MEK-1 mutant and IiOVA (MEK1 AA–IiOVA) before induction of AIA, destruction of articular cartilage and bone is evident (**arrowheads**), with extensive cellular infiltration (**arrows**). Original magnification × 40. **B** = trabecular bone; **C** = articular cartilage; **AC** = articular capsule. **B**, Histologic scores were determined to measure the extent of joint destruction and inflammation after lentivector vaccination. Bars show the mean ± SEM results in 5 mice per group. ∗∗∗ = highly significant difference (*P* < 0.001). **C**, Left, ERK-activated immature dendritic cells (iDC) present antigen under suboptimal conditions to T cells, leading to reduced CD8 T cell expansion. Transforming growth factor β (TGFβ) drives antigen-specific Treg cell differentiation rather than expansion of effector CD4+ T cells. Right, A second antigen exposure under inflammatory conditions drives strong Treg cell proliferation, with antigen-specific suppressive activities. MHC-I = class I major histocompatibility complex.

Histopathologic studies confirmed that direct administration of the ERK activator and IiOVA protected joints from inflammation and bone destruction after intraarticular challenge, when OVA was provided with mBSA (Figure 6A). In contrast, when the ERK inhibitor was administered instead, articular cartilage/bone destruction and extensive cellular infiltration in the articular capsule were evident, together with a significant increase in the arthritis score (Figures 6A and B).

## DISCUSSION

To our knowledge, this is the first time that selective activation of a single intracellular signaling pathway has been used to establish antigen-driven immunosuppression that effectively inhibits inflammatory arthritis. We have demonstrated that the ERK pathway is suppressive, by using a specific endogenous activator rather than MEK chemical inhibitors. Simultaneous ERK activation and antigen expression inhibited effector T cell responses, leading to antigen-specific FoxP3+ Treg cell differentiation. These Treg cells strongly expanded in vivo after a second antigen encounter in inflammatory conditions, and exerted inhibitory activities in the presence of the specific class II epitope peptide. These results support a mechanism in which antigen recognition by Treg cells is required or, at least, highly enhances their suppressive activity (3). Interestingly, rechallenge with OVA in the absence of adjuvant also resulted in efficient expansion of FoxP3+ Treg cells independent of the vaccination regimen with lentivectors. It is tempting to speculate that this Treg cell expansion could be the underlying mechanism by which administration of purified proteins in the absence of adjuvants results in tolerance, as opposed to immunization.

Direct lentivector administration of the ERK activator resulted in strong immunosuppression, even though the lentivectors were not per se specifically targeted to DCs. However, we and others have shown that subcutaneous lentivector vaccination transduces skin-derived DCs, which migrate to draining lymph nodes and present antigens to T cells (34,35). Accordingly, our findings showed that ERK activation in BMDCs resulted in generation of immunosuppressive DCs, which also inhibited inflammatory arthritis. Interestingly, ERK inhibition also increased the immunogenicity of the lentiviruses and DCs. In vivo, the ERK-dependent DC suppressive activity, including FoxP3+ T cell differentiation, relied completely on DC-derived TGFβ, but not on IL-10. Many studies using MEK chemical inhibitors have linked ERK activation with IL-10 secretion (39). However, we have never observed increased IL-10 production after selective ERK activation in DCs (25). Furthermore, unlike the indispensable role of TGFβ, we found that DC-derived IL-10 was not needed in order for ERK-dependent immunosuppression to occur.

On the basis of our results, we propose a model for ERK tolerogenic activity (Figure 6C). In the first stage, ERK activation keeps DCs in an immature stage. Suboptimal antigen presentation combined with increased secretion of bioactive TGFβ inhibits CD8 T cell expansion and leads to Treg cell differentiation. In the second stage, these Treg cells strongly proliferate after antigen reencounter in inflammatory conditions, thus inhibiting inflammatory T cell responses.

Importantly, selective ERK activation differentiated regulatory human PBMC–derived DCs that had the capacity to expand functional FoxP3+ Treg cells, which could be used to suppress autoimmune arthritis in humans. ERK-activated human PBMC–derived DCs exhibited an immature phenotype, with high levels of bioactive TGFβ secretion. ERK-activated human PBMC–derived DCs significantly induced differentiation of CD4+CD25^high^FoxP3+ T cells from allogeneic naive T cells. Functional human FoxP3+ Treg cells are restricted to the CD25^high^ population (40), and in this study, we demonstrated their capacities to inhibit proliferation of activated CD8 T cells. The mechanisms by which these induced Treg cells inhibit CD8 T cell proliferation in the presence of ERK-activated human PBMC–derived DCs are currently under investigation. These results indicate that selective ERK activation could be applied for the treatment of autoimmune arthritis in humans. Accordingly, experimental inflammatory arthritis was effectively inhibited in a mouse model. Moreover, even though antigen (OVA)–driven immunosuppression was achieved, we demonstrated that direct targeting of the arthritogenic antigen (mBSA) was not required. This could be of particular importance because arthritogenic antigens are not well characterized and may vary between patients.

Most cellular-based therapies rely on ex vivo modification of large numbers of cells from the patients. These strategies are promising, although they may be cumbersome and expensive (4,41). In this study, direct lentivector administration of the ERK activator resulted in an effective, straightforward protocol with a significant improvement over strategies involving DC or T cell vaccination.
